# Low meniscus reoperation rates following meniscus repair during anterior cruciate ligament reconstruction in Turkey: an in-depth national analysis of 8-years

**DOI:** 10.1186/s12891-024-07662-0

**Published:** 2024-07-17

**Authors:** Izzet Bingol, Saygin Kamaci, Ibrahim Kaya, Ali Aykut Fidanci, Mustafa Okan Ayvali, Naim Ata, M. Mahir Ulgu, Suayip Birinci, Ozgur Ahmet Atay, Alper Kaya

**Affiliations:** 1 Faculty of Medicine, Department of Orthopedics and Traumatology, Ankara Yildirim Beyazit University, Ankara, Türkiye; 2 Department of Orthopaedic Surgery, Hacettepe University, Ankara, Türkiye; 3 Department of Orthopaedics and Traumatology, Dr. Abdurrahman Yurtaslan Ankara Oncology Training and Research Hospital, Ankara, Türkiye; 4 General Directorate of Health Information Systems, Ankara, Türkiye; 5 Ministry of Health, General Directorate of Health Information Systems, Ankara, Türkiye; 6 Ministry of Health, Deputy Minister, Ankara, Türkiye; 7 Department of Orthopaedic Surgery, Acibadem University, Istanbul, Türkiye

**Keywords:** Anterior cruciate ligament reconstruction, Concomitant meniscal injury, Meniscus repair, Meniscectomy, Meniscus repair failure

## Abstract

**Background:**

Concomitant knee injuries, such as meniscal tears, are observed in up to 80% of cases and can have a detrimental impact on outcomes following anterior cruciate ligament reconstruction (ACLR). Over recent decades, there has been a growing recognition of the importance of preserving meniscal tissue. Consequently, the prevalence of meniscal-preserving procedures has been on the rise.

**Purpose:**

The objective of this study was to examine the prevalence of concurrent meniscal procedures, assess the success rate, and identify factors associated with the failure of meniscal repair in patients undergoing ACLR.

**Methods:**

All patients who underwent ACLR due to anterior cruciate ligament (ACL) injury between January 2015 and December 2022 were extracted from the Republic of Türkiye National health system using operation-specific procedure codes. Patients with multiple ligament injuries, revision ACL patients, and patients with missing data were excluded from the study. The treatment methods were grouped into the subsets of meniscectomy, meniscal repair, transplantation, and meniscectomy + repair. The distribution of ACLR and meniscus treatment methods according to years, age and sex groups, hospital characteristics, and geographical regions was examined. A secondary analysis was performed to assess the effect of patient demographics and hospital healthcare level on revision meniscal procedures in the ACLR + concomitant meniscal repair group.

**Results:**

A total of 91,700 patients who underwent ACLR between 2015 and 2022 were included in the study. A concomitant meniscal procedure was noted in 19,951(21.8%) patients (16,130 repair,3543 meniscectomy). In the 8 years studied, meniscus repair rates increased from 76.3%to87.9%, while meniscectomy rates decreased from 23.7%to12.1% (*p* < 0.001). The revision meniscus surgery rate following ACLR + meniscal repair was 3.7%at a mean follow-up of 50 ± 26 months. The interval between primary and revision surgery was 20.5 ± 21.2 months. The meniscectomy rates were higher in community hospitals, while private hospitals showed the lowest revision meniscus surgery rates. Younger age was associated with increased meniscus repair failure rates.

**Conclusion:**

The propensity towards using repair techniques to treat meniscal tears during concurrent ACLR has significantly increased in Turkey. Age and the healthcare level of the treating hospital affect the success of meniscal repair.

## Introduction

Meniscal tears occurring alongside anterior cruciate ligament (ACL) injuries are prevalent, seen in up to 80% of cases, and can adversely impact the outcomes following anterior cruciate ligament reconstruction [1–8]. Such tears are recognized as a risk factor for osteoarthritis and are crucial in determining the long-term prognosis for this condition [8, 9]. Studies have indicated that both partial and complete meniscectomies are significantly associated with degenerative changes in the joint [10–13]. Long-term clinical outcome studies have demonstrated notably superior outcomes when the meniscus remains intact or is repaired during ACL reconstruction (ACLR) [13–16]. Consequently, the importance of preserving meniscal tissue has gained considerable recognition in recent decades, leading to an increase in the prevalence of meniscal-preserving procedures [6, 13, 15].

Various factors have been proposed to influence the failure rates of meniscal repair, including patient age, sex, body mass index (BMI), location of meniscal injury (medial/lateral), cartilage injury, associated ACLR, meniscal repair technique, ACLR technique, and time from injury to surgery [2, 4, 5, 17–23]. Additionally, geographic factors such as hospital characteristics and surgeons' volume may also play a role in surgical outcomes and revision rates. Therefore, it is imperative to analyze nationwide data to evaluate treatment trends and identify local factors associated with failure rates. This comprehensive analysis allows for a better understanding of the factors influencing meniscal repair outcomes and informs strategies for improving patient care and surgical outcomes.

The objective of this study was to analyze the prevalence of concurrent meniscal procedures, revision meniscal procedure rates, and factors contributing to meniscal repair failure in patients undergoing anterior cruciate ligament reconstruction, utilizing data from the Turkish National Personal Health Record System database. We hypothesized that meniscal repair is more commonly performed in younger patients and that its incidence is increasing across different geographic regions in Turkey. Additionally, we aimed to assess how the success rate of meniscal repair is influenced by patient age and the characteristics of the hospital where the primary surgery was performed.

## Materials & methods

The health records of individuals who underwent primary ACLR due to ACL injury in public, private, and university health institutions were obtained from the e-health database of the Ministry of Health of the Republic of Turkey [24]. The study was conducted in accordance with the Declaration of Helsinki and approval was obtained from the Republic of Türkiye Ministry of Health with a waiver for informed consent for retrospective data analysis (ID: 95741342-020/27112019).

All patients who underwent ACLR (surgery codes: 612,830, P612830) due to ACL injury between January 2015 and December 2022 were determined from the Republic of Türkiye National health system using operation-specific procedure codes (https://skrs.saglik.gov.tr/). Patients with multiple ligament injuries, revision ACL patients, and patients with missing data were excluded from the study, and only those who underwent primary ACLR were included. After the patients who had undergone ACLR were determined, the treatment method applied to concomitant meniscal injury according to the procedure codes used was grouped into the subsets of meniscectomy (surgery codes: 613,160, P613160), meniscal repair (surgery codes: 612,760, P612760), and transplantation (surgery codes: 612,770, P612770). Patients without meniscus injury were grouped as isolated ACLR. Patient demographics (age, sex, and BMI) were obtained from the e-health database after a valid patient pool was created. The distribution of ACLR and meniscus treatment method according to years, age (< 18 years, 18–29 years, 30–39 years, 40–49 years, 50–59 years, 60–69 years, and > 70 years), sex, hospital characteristics (private, community hospitals, and university hospitals), and geographical regions were analyzed. The reason for investigating the hospital characteristics was to identify the effect of subspecialty training on re-operation rates. Fellowship trained sports medicine surgeons are located in university and private hospitals in Turkey. Additionally, university hospitals serve as teaching hospitals for orthopedic residents. BMI was classified as underweight and normal (≤ 24.9 kg/m^2^), overweight (25.0–29.9 kg/m^2^), and obese (≥ 30.0 kg/m^2^) in patients with available records.

The ACLR + meniscus repair group was analyzed to assess the occurrence of revision meniscus surgery (utilizing meniscectomy or meniscus repair surgery codes) on the same knee. Patients were categorized into two groups: isolated meniscus revision and meniscus revision + revision ACLR. Factors potentially associated with revision meniscus surgery, including age group, sex, BMI, and characteristics of the hospital where the primary surgery was performed, were examined and analyzed.

### Statistical analysis

SPSS 25 (Armonk, NY, USA: IBM Corp.) was used in our study. Frequency and percentage statistics were used for descriptive measures. Chi-squared tests (Pearson) were used for categorical variables. The Cochran–Armitage test for trend was used to analyze yearly proportions of concomitant meniscal surgery types. Univariate logistic regression was used to determine whether the BMI group posed a risk for meniscal injury. Since the categories of the meniscus injury outcome variable were unevenly distributed, this situation was resolved with the RStudio 2023.03.0 ROSE v0.0 package. The significance level for all tests was 0.05.

## Results

**Fig. 1:**
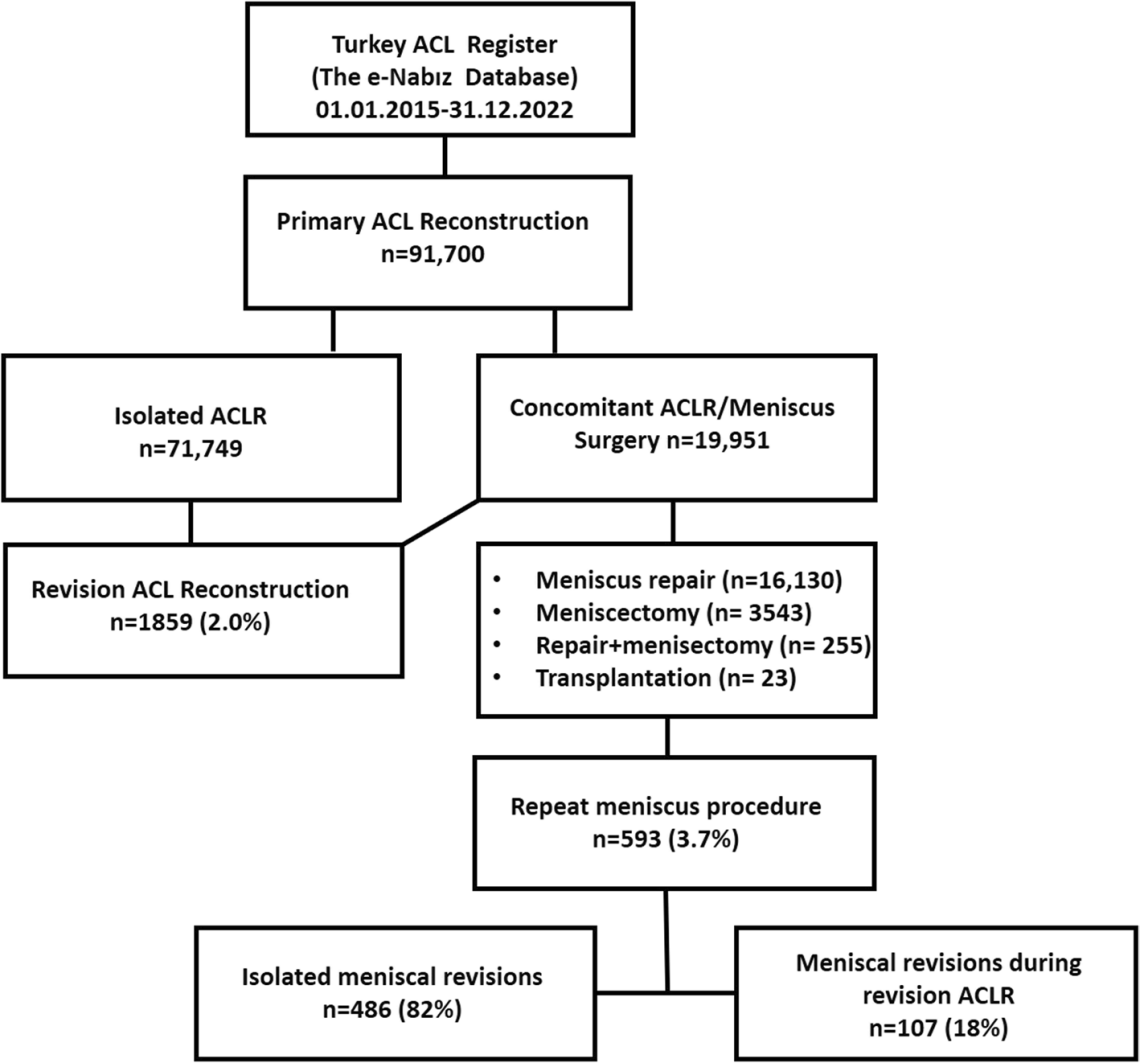
Flow diagram of patients who underwent primary ACLR and concomitant ACLR/meniscus surgery, defined between 2015 and 2022

The study included a total of 91,700 patients who underwent ACLR between 2015 and 2022 (Fig. 1). The dataset predominantly comprised male patients (88.6%), with a notable concentration in the 18–29 years (48.4%) and 30–39 years (30.6%) age groups, as well as in the underweight and normal weight BMI group (44%). Among the patients who underwent ACLR, concomitant treatment for meniscal injury was administered to 19,951 individuals (21.8%). These procedures included 16,130 (80.8%) meniscal repairs, 3543 (17.8%) meniscectomies, 255 (1.3%) repair + meniscectomies, and 23 (0.1%) transplantations (Table 1). Due to the low number of patients who underwent meniscus transplantation and repair + meniscectomy, they were excluded from the statistical evaluation. The transplantation code encompassed meniscus transplantation from fresh frozen cadaver tissue and meniscus scaffold augmentations, which are infrequently utilized in Turkey due to their high costs.

**Table 1:** Baseline statistics for ACLR

Variables	Categories	n	%
Age groups	< 18	4892	5.3%
	18–29	44,389	48.4%
	30–39	28,051	30.6%
	40–49	12,226	13.3%
	50–59	2142	2.3%
Sex	Male	81,201	88.6%
	Female	10,499	11.4%
Geographic region	Marmara	25,955	28.3%
	Aegean	9375	10.2%
	Mediterranean	14,602	15.9%
	Central Anatolia	16,449	17.9%
	Black Sea	9462	10.3%
	Eastern Anatolia	4584	5.0%
	Southeastern Anatolia	11,273	12.3%
Hospital status	University	12,465	13.6%
	Community	48,853	53.3%
	Private	30,382	33.1%
Meniscus surgery	Repair	16,130	80.8%
	Meniscectomy	3543	17.8%
	Repair + Meniscectomy	255	1.3%
	Transplantation	23	0.1%
BMI	≤ 24.9	9105	44.0%
	25–29.9	8019	38.8%
	≥ 30	3548	17.2%

Categorical variables were expressed as frequency (percentage)

In patients under 18 years of age, concomitant meniscus treatment was proportionally higher compared to other age groups (*p* = 0.001). Specifically, meniscus repair was performed in 90.8% of patients under 18 years old, in 82.3% of those aged 18–29 years, and in 78.9% of those aged 30–39 years. Therefore, meniscus repair was utilized more frequently in younger patients (*p* < 0.001). Among the patients, meniscus repair was employed in 81.6% of men and 85.2% of women (*p* < 0.001). Meniscectomy was more frequently performed (21.1%) in obese patients, while meniscal repair (84.7%) was more common in underweight and normal weight patients (Table 2). Upon univariate modeling of the BMI values of 20,672 patients, according to the meniscus injury outcome variable, the injury risk in overweight patients compared to underweight and normal weight patients was calculated as OR = 1.069 (95% CI: 1.009–1.131, *p* = 0.020). However, obese patients did not exhibit a higher risk of meniscus injury (OR = 0.945, 95% CI: 0.868–1.028, *p* = 0.192). The hospital characteristics and geographic distribution of ACLR and meniscal procedures across Turkey are detailed in Table 1 and Table 2.

**Table 2:** Evaluations according to the meniscus treatment method

Variables	Categories	Meniscus repair n (%)	Meniscectomy n (%)	p
Age group	< 18	1071 (90.8)	100 (8.5)	0.000
	18–29	8069 (82.3)	1616 (16.5)	
	30–39	4798 (78.9)	1174 (19.3)	
	40–49	1892 (75.4)	575 (22.9)	
	50–59	300 (78.1)	78 (20.3)	
Sex	Male	14,391 (81.6)	3242 (18.4)	< 0.001
	Female	1739 (85.2)	301 (14.8)	
BMI	≤ 24.9	1597 (84.7)	275 (14.6)	0.000
	25–29.9	1394 (80.8)	303 (17.6)	
	≥ 30	519 (77.2)	142 (21.1)	
Geographic region	Marmara	4419 (79.9)	1115 (20.1)	< 0.001
	Aegean	1215 (67.2)	592 (32.8)	
	Mediterranean	1613 (74.3)	557 (25.7)	
	Central Anatolia	3797 (89.4)	449 (10.6)	
	Black Sea	1308 (89.2)	158 (10.8)	
	Eastern Anatolia	1207 (83.9)	231 (16.1)	
	Southeastern Anatolia	2571 (85.4)	441 (14.6)	
Hospital status	University	3701 (91.7)	335 (8.3)	< 0.001
	Community	8972 (75.3)	2940 (24.7)	
	Private	3457 (92.8)	268 (7.2)	

Categorical variables were expressed as frequency (percentage)

**Fig. 2:**
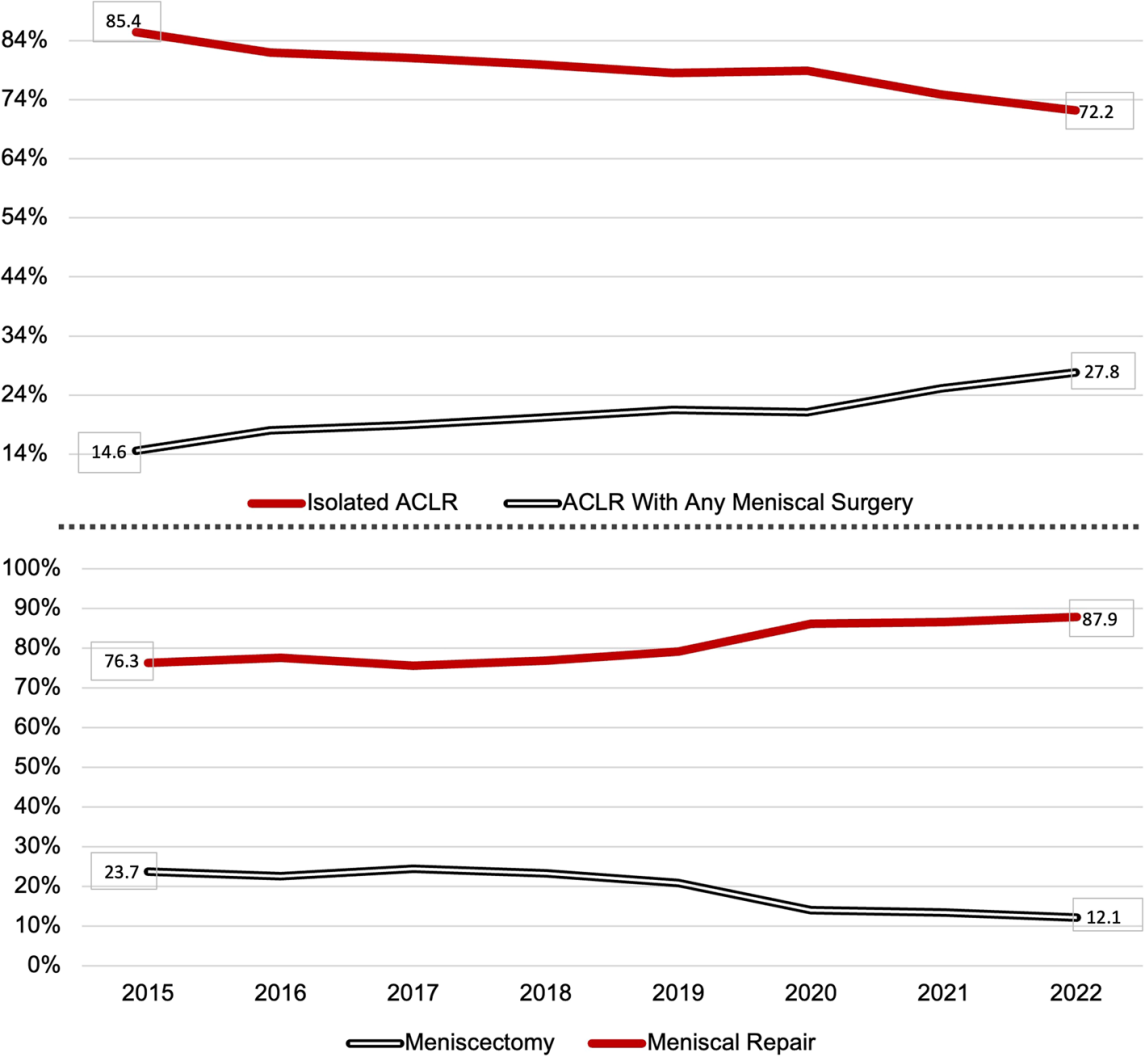
Change in isolated ACLR and concomitant meniscus surgery rates over the years

Despite a slight decrease in ACLRs during the COVID-19 pandemic in 2020 and 2021, there was a general increasing trend observed between 2015 and 2022. Although isolated ACLR rates experienced a decline, there was an overall rise in meniscal procedures performed during ACLR. Over the 8-year study period, the rates of meniscus repair increased from 76.3% to 87.9%, while meniscectomy rates decreased from 23.7% to 12.1% (*p* < 0.001) (Fig. 2). The rate of meniscus repair versus meniscectomy was significantly higher in private hospitals and university hospitals compared to community hospitals (92.8%, 91.7%, and 75.3%, respectively) (*p* < 0.001).

In the ACLR + meniscus repair group comprising 16,130 patients, the mean follow-up duration was 50 ± 26 months. The rate of revision meniscus surgery was 3.7% (593 patients). Among these, 18% (107 patients, 0.67% of the study cohort) underwent concomitant revision ACLR. The average time between primary and revision surgery was 20.5 ± 21.2 months. The rate of meniscal revision was similar between males and females (3.7% male versus 3.4% female). Notably, the pediatric group (5.9%) and the age group of 18–29 years (4%) demonstrated significantly higher meniscus repair failure rates compared to other age groups (*p* = 0.000). Among the 3510 patients with available BMI data, there was no significant difference in revision meniscal surgery rates between underweight and normal weight, overweight, and obese patients (4.3% vs. 4.6% vs. 2.9%, respectively) (*p* = 0.248) (Table 3).

**Table 3:** Evaluation of the rate of revision meniscus surgery according to the variables in patients who underwent meniscus repair

Variables	Categories	Revision yes n (%)	Revision no n (%)	p
Age groups	< 18	63 (5.9)	1008 (94.1)	0.000
	18–29	325 (4.0)	7744 (96.0)	
	30–39	145 (3.0)	4653 (97.0)	
	40–49	52 (2.7)	1840 (97.3)	
	50–59	8 (2.7)	292 (97.3)	
Sex	Male	534 (3.7)	13,857 (96.3)	0.506
	Female	59 (3.4)	1680 (96.6)	
Total		**593 (3.7)**	**15,537 (96.3)**	
BMI	≤ 24.9	69 (4.3)	1528 (95.7)	0.248
	25–29.9	64 (4.6)	1330 (95.4)	
	≥ 30	15 (2.9)	504 (97.1)	
Total		148 (4.2)	3362 (95.8)	

Categorical variables were expressed as frequency (percentage)

**Fig. 3:**
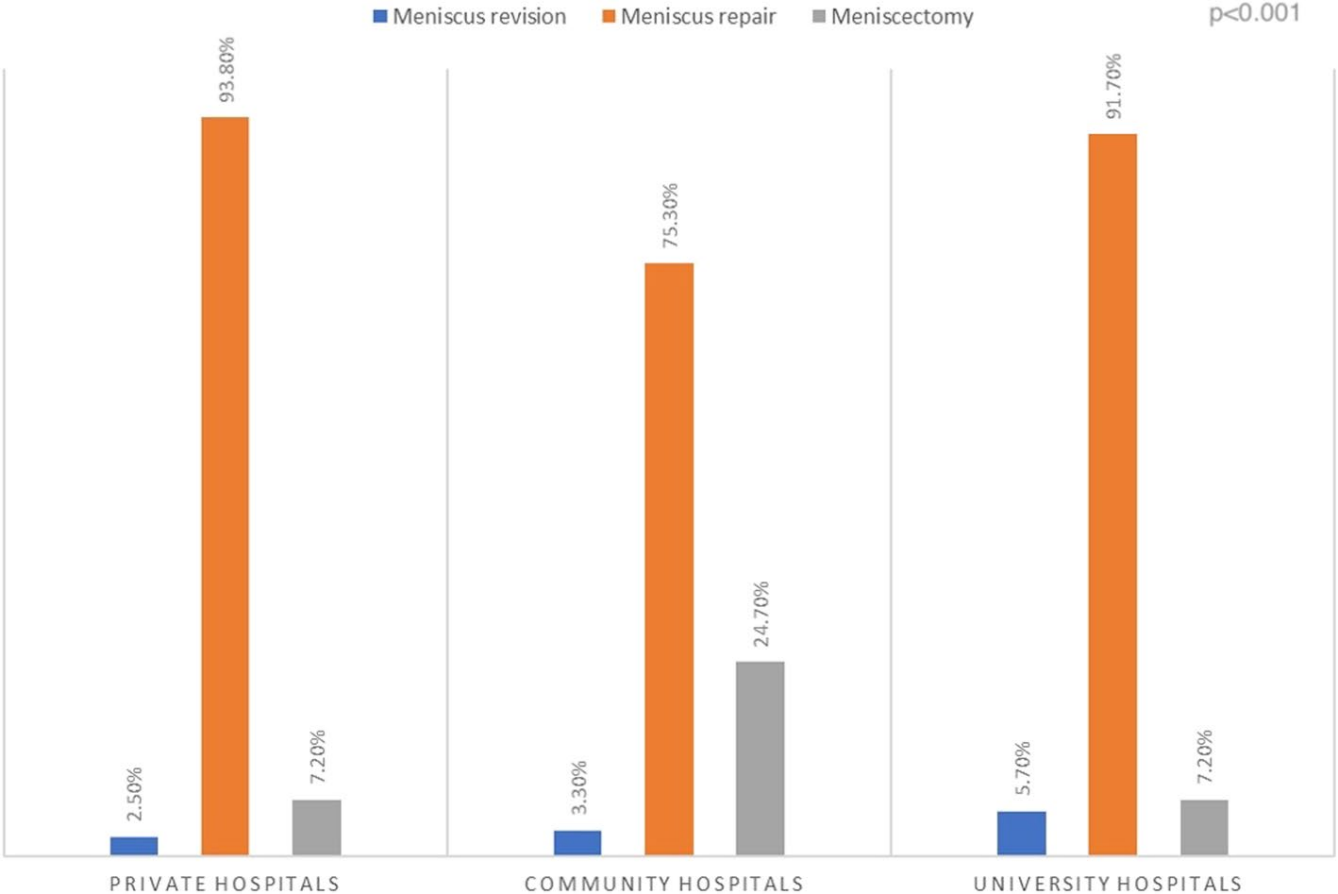
Meniscus treatment method and meniscus revision rates according to hospital status

The characteristics of the hospital where the primary surgery was performed significantly influenced meniscus repair failure rates. While the mean follow-up time was similar across hospital groups, there were notable differences in the distribution of age groups. The < 18 years and 18–29 years age groups, which exhibited the highest meniscal revision rates, were predominantly treated in community hospitals, followed by private hospitals and university hospitals (54.2%, 31.1%, and 24.7%, respectively) (*p* = 0.00). Community hospitals demonstrated the highest rates of meniscectomy (24.7%). In contrast, private hospitals exhibited the lowest rates of meniscal revision surgery compared to community hospitals and university hospitals (2.5%, 3.3%, and 5.7%, respectively) (*p* = 0.00) (Fig. 3).

## Discussion

The most important finding of this study was ACLR + concomitant meniscal repair showed notably low reoperation rates in the mid-term follow-up. Among patients who underwent ACLR between 2015 and 2022, 21.8% received concomitant meniscus procedures. Our findings reveal that younger age significantly heightens the risk of requiring revision meniscus surgery, while sex and BMI appear to exert no discernible influence on this outcome.

he trend of increasing rates of meniscus repair observed in our study, from 76.3% in 2015 to 87.9% in 2022, is consistent with findings from other population-based studies [4, 25, 26]. Parker et al., utilizing data from the American Board of Orthopedic Surgery database spanning from 2004 to 2012, reported a similar trend. They noted a significant rise in the annual rates of isolated meniscus repair compared to meniscectomy rates over the study period. Specifically, they observed a 56% increase in meniscus repair rates and an 18% increase in meniscectomy rates combined with ACLR over the years [3]. These findings underscore the evolving treatment landscape favoring meniscus preservation strategies, which aligns with current literature trends.

The prevalence of meniscal tears accompanying ACL injury, as reported in the literature, typically ranges from approximately 47% to 73% [27–34]. However, in our study, the rate of concomitant meniscal operations in patients undergoing ACLR was found to be 21.8%, which is notably lower than the overall incidence reported in the literature. There are several possible explanations for this discrepancy. It's possible that not all meniscal tears are diagnosed or that some tears are managed conservatively without surgical intervention, thereby not being captured in our analysis. Additionally, some tears may not be treated surgically but instead monitored over time. However, when specifically analyzing meniscal injuries requiring repair within our 8-year ACLR cohort, we found that the percentage was 17.6%, which is more comparable to the results of previously published national database studies. This suggests that the rate of meniscal injuries requiring repair in our study aligns more closely with existing literature [1, 4, 6, 32]. It's noteworthy that the rate of meniscectomies during ACLR in our study was very low at 0.6%. This contrasts with findings from the literature and raises the possibility that these procedures might be underreported or coded differently, such as arthroscopic debridement, by surgeons. Unfortunately, the underlying reasons for this discrepancy could not be determined in our analysis.

Increased BMI has been consistently associated with higher rates of concomitant meniscal injury and meniscectomy, particularly among adolescents [35–38]. However, in our study, we observed a positive correlation between BMI and the use of meniscectomy, indicating that a higher BMI was indeed associated with an increased likelihood of undergoing meniscectomy. Interestingly, despite the association between BMI and meniscectomy rates, obesity itself did not appear to be a significant risk factor for meniscal injury in our analysis. This finding suggests that while higher BMI may increase the likelihood of requiring meniscectomy once a meniscal injury occurs, it may not necessarily predispose individuals to a higher risk of sustaining such injuries in the first place. Moreover, our study found no significant difference in revision meniscal surgery rates among patients with different BMI levels. This aligns with findings from a previous systematic review by Yeo et al., which reported similar rates of meniscal repair failure between patients with low and high BMI [21]. Taken together, these findings suggest that while BMI may influence the type of treatment required for meniscal injuries, it may not significantly impact the success rates of meniscal repair procedures.

Meniscus repair performed concurrently with ACLR has been associated with lower failure rates compared to isolated meniscus repair procedures [21]. Previous studies have reported success rates ranging from 75 to 92% for meniscus repair performed during ACLR [2, 17, 18, 23]. A systematic review by Paxton et al. found a 14% revision rate for meniscal repairs performed concomitantly with primary ACLR [19]. Similarly, an analysis of New Zealand's ACL registry reported a 6.6% meniscectomy rate following ACLR combined with meniscus repair at a mean follow-up of 2.9 years ref. Similarly, an analysis of New Zealand's ACL registry reported a 6.6% meniscectomy rate following ACLR combined with meniscus repair at a mean follow-up of 2.9 years [20].

The majority of the meniscus revision procedures (82%) observed in our study were isolated meniscus operations, particularly in younger patients. The average time between the primary and revision surgery in our study was 20.5 ± 21.2 months, which is consistent with findings in the literature. Studies have shown that meniscus re-tears tend to occur within the first few years following anterior cruciate ligament reconstruction (ACLR), with younger patients and those engaging in high levels of physical activity being at greater risk [35–38]. Rahardja et al. showed increased rates of re-tear causing meniscectomy in patients aged 21–30 years for medial meniscus and patients younger than 20 years old for lateral meniscus following ACLR with meniscus repair [7]. imilarly, our results indicated significantly increased rates of revision meniscal surgery in patients younger than 18 years and those aged 19–29 years old.

Surgeon experience and hospital characteristics are indeed crucial factors that can influence the rate of meniscal re-tears and re-operations following ACLR combined with meniscus repair. The New Zealand ACL registry study claimed that procedures performed by low volume surgeons (< 30 cases/year) displayed 1.8 times increased meniscus failure rates compared to those of high volume surgeons [7]. Moreover, research has demonstrated that better patient outcomes are achieved when ACLR surgery is performed by surgeons with subspecialty training in sports medicine, as opposed to general orthopedic practitioners [5, 7, 22]. n our study, we observed increased rates of re-operation in community hospitals and university hospitals compared to private hospitals. This trend may be attributed to the composition of the surgical staff in these settings. Community hospitals in Turkey often employ general orthopedic surgeons rather than sports medicine specialists with subspecialty training. Consequently, the absence of subspecialty training among the surgical staff in community hospitals may contribute to the increased rates of re-operation observed in our study, aligning with findings in the existing literature.

The role of sex in meniscus repair failure rates following anterior cruciate ligament reconstruction (ACLR) combined with concomitant meniscus repair has been the subject of considerable investigation. While some studies have reported increased reoperation rates in females following ACLR, others have found no significant difference in meniscal repair failure rates between males and females [39]. In our study, we similarly found no significant effect of sex on meniscus repair failure rates following ACLR combined with concomitant meniscus repair. These findings are consistent with several national database studies that have failed to demonstrate a clear association between sex and meniscal repair outcomes in the context of ACLR.

The present study offers valuable insights into the prevalence and outcomes of meniscal procedures performed in conjunction with anterior cruciate ligament reconstruction (ACLR) using a large national database. However, it is important to acknowledge several limitations inherent to the study design. Firstly, as with many national database studies, the data primarily capture information on surgically treated patients, potentially excluding non-surgical cases and limiting the generalizability of the findings to the broader population of ACL-injured individuals. Secondly, the study lacks detailed information on factors such as surgical techniques, specific types and locations of meniscal tears, accompanying joint pathologies, and clinical outcomes. These factors could have significant implications for the success rates of meniscal procedures and should be considered in future research. Furthermore, the reliance on diagnostic and procedural codes for data collection introduces the potential for errors in coding and billing, which may affect the accuracy of the results.

## Conclusion

The study highlights the high survival rate of meniscus repair performed concurrently with ACLR in the mid-term follow-up, with a 96.3% success rate. However, it also identifies younger age as a factor associated with decreased success of meniscal repair, while sex and BMI were not found to have a significant effect on revision rates. These findings underscore the importance of further detailed analysis to elucidate the factors influencing meniscal healing rates. Future research in this area could focus on exploring additional variables that may impact the success of meniscal repair, such as the type and location of meniscal tears, surgical techniques, rehabilitation protocols, and patient-specific factors such as activity level and comorbidities.

## Data Availability

The datasets used and/or analysed during the current study are available from the corresponding author on reasonable request.
